# Transforming Growth Factor-β3/Recombinant Human-like Collagen/Chitosan Freeze-Dried Sponge Primed With Human Periodontal Ligament Stem Cells Promotes Bone Regeneration in Calvarial Defect Rats

**DOI:** 10.3389/fphar.2021.678322

**Published:** 2021-04-23

**Authors:** Shiyi Huang, Fenglin Yu, Yating Cheng, Yangfan Li, Yini Chen, Jianzhong Tang, Yu Bei, Qingxia Tang, Yueping Zhao, Yadong Huang, Qi Xiang

**Affiliations:** ^1^ Institute of Biomedicine and Guangdong Provincial Key Laboratory of Bioengineering Medicine, Jinan University, Guangzhou, China; ^2^ Biopharmaceutical R and D Center of Jinan University, Guangzhou, China; ^3^ Department of Stomatology, Jinan University Medical College, Guangzhou, China

**Keywords:** periodontal ligament stem cells, transforming growth factor 3, stem cell therapy, skull bone defect repair, freeze-dried sponge

## Abstract

Patients with a skull defect are at risk of developing cerebrospinal fluid leakage and ascending bacterial meningitis at >10% per year. However, treatment with stem cells has brought great hope to large-area cranial defects. Having found that transforming growth factor (TGF)-β3 can promote the osteogenic differentiation of human periodontal ligament stem cells (hPDLSCs), we designed a hybrid TGF-β3/recombinant human-like collagen recombinant human collagen/chitosan (CS) freeze-dried sponge (TRFS) loading hPDLSCs (TRFS-h) to repair skull defects in rats. CFS with 2% CS was selected based on the swelling degree, water absorption, and moisture retention. The CS freeze-dried sponge (CFS) formed a porous three-dimensional structure, as observed by scanning electron microscopy. In addition, cytotoxicity experiments and calcein-AM/PI staining showed that TRFS had a good cellular compatibility and could be degraded completely at 90 days in the implantation site. Furthermore, bone healing was evaluated using micro-computed tomography in rat skull defect models. The bone volume and bone volume fraction were higher in TRFS loaded with hPDLSCs (TRFS-h) group than in the controls (*p* < 0.01, *vs.* CFS or TRFS alone). The immunohistochemical results indicated that the expression of Runx2, BMP-2, and collagen-1 (COL Ⅰ) in cells surrounding bone defects in the experimental group was higher than those in the other groups (*p* < 0.01, *vs.* CFS or TRFS alone). Taken together, hPDLSCs could proliferate and undergo osteogenic differentiation in TRFS (*p* < 0.05), and TRFS-h accelerated bone repair in calvarial defect rats. Our research revealed that hPDLSCs could function as seeded cells for skull injury, and their osteogenic differentiation could be accelerated by TGF-β3. This represents an effective therapeutic strategy for restoring traumatic defects of the skull.

## Introduction

Craniocerebral injury is a global public health concern. In Australia, there are approximately 338,700 people with disabilities related to craniocerebral injuries (1.9% of the population). Large-scale skull defects are very difficult to repair and are associated with the risk of cerebrospinal fluid leakage and ascending bacterial meningitis, causing approximately 30% of deaths each year in the United States (Gardner and Zafonte, 2016). Survivors can have a variety of residual symptoms that affect their cognition, movement, and sensation (Xu et al., 2015). Autologous bone transplantation is a widely recognized clinical treatment method. However, sources of autologous bone are limited, and the required bone needs to be removed from other parts of the patient’s body, increasing the risk of infection during surgery. In recent years, stem cell therapy has rapidly developed in bone defect repair. Bioactive materials loaded with mesenchymal stem cells (MSCs) are the most promising materials for the treatment of bone injuries.

**GRAPHICAL ABSTRACT:**
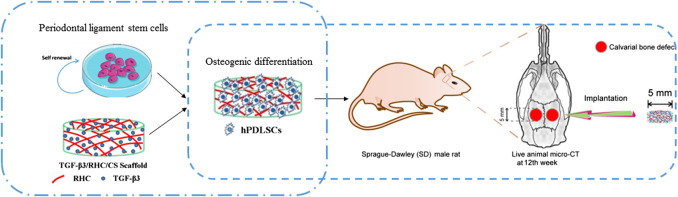


Stem cells have ability to induce tissue regeneration and repair areas of injury (Zhang et al., 2015a). Among them, MSCs have strong application prospects in bone repair therapy because of their ability to self-replicate and to undergo multi-directional differentiation. Bone marrow mesenchymal stem cells (BMSCs), adipose mesenchymal stem cells, and umbilical cord mesenchymal stem cells (UC-MSCs) are examples of MSCs that are commonly used in bone repair therapy (Zhang et al., 2018). Human periodontal ligament stem cells (hPDLSCs), a type of MSC from the periodontal tissue of adult third molars, are considered to be relatively suitable MSC sources for bone tissue regeneration (Chalisserry et al., 2017). More interestingly, the acquisition of hPDLSCs is relatively simple, convenient, and less invasive, and some discarded teeth in clinical surgery can be used as a reliable source of stem cell banks (Luo et al., 2021). Zhang et al. (2018) reported that hPDLSCs and BMSCs exerted similar cell behaviors, both having the ability to induce colony formation and multi-directional differentiation. Thus, these stem cells could be promising potential seed cell population types for use in cell-based therapy and tissue regeneration (Luo et al., 2018). Recently, Professor Yan Wang and her laboratory fabricated zein/gelatin/nano-hydroxyapatite nanofiber membranes to load hPDLSCs, which is an effective strategy for the treatment of periodontitis in the future, as part of their research on odontogenic cells (Yang et al., 2017). Diomede et al. found that the combination of hPDLSCs and collagen membranes can effectively promote the repair of rat skull defects (Trubiani et al., 2019). Our group explored the osteogenic differentiation of hPDLSCs *in vitro* for applications in bone repair (Li et al., 2019; Li et al., 2020). Many studies have shown that hPDLSCs can maintain multi-differentiation performance in long-term culture, and are potentially ideal tissue-engineering seed cells (Ou et al., 2018). In summary, hPDLSCs can transfer generations many times *in vitro* while maintaining stemness, making them suitable for broad applications in bone injury repair.

Over the last decade, an increasing number of studies have focused on the development of natural, biodegradable, and biocompatible nanocomposites for bone replacement stem cell therapy. An ideal bio-nanocomposite should possess not only good mechanical properties but also an architecture that mimics the *in vitro* extracellular matrix (ECM) (Cheng et al., 2020). It provides a suitable habitat for stem cells and promotes the differentiation of stem cells to achieve tissue regeneration (Ma et al., 2020). Chitosan and collagen are the most widely used biological matrices owing to their biocompatibility and biodegradability. In particular, collagen is the most abundant matrix in the ECM and plays a decisive role in cell adhesion, migration, and differentiation, as well as promotes the growth of new tissues. However, the biodegradation rate and low mechanical strength of a single collagen-based or CS-based scaffold are crucial problems that limit their applications. The main obstacle in the wide use of collagen or CS alone is their almost complete lack of solubility in water and alkaline solutions. To overcome this obstacle, we united modified collagen and CS to achieve a relatively ideal scaffold material for bone repair and bone tissue engineering. Ramkumar et al. combined MSCs with a CS-collagen matrix to form a modular microstructure and repair critical skull defects in rats (Annamalai et al., 2019). Natural collagen is difficult to prepare and the degradation rate cannot be controlled, while recombinant human-like collagen (RHC) redesigns and optimizes the main functional sequence of natural collagen, and the optimized gene sequence is reconstructed and expressed to obtain a new protein with collagen properties (Zhang et al., 2015b). In the last three decades, pioneering experiments have led to the expression of recombinant human or human-like collagens, providing a foundation for studies on the potential use of these proteins as substitutes for animal-derived collagens (Sionkowska et al., 2020). At present, RHC scaffold materials have been used in regenerative medicine, for example, as blood vessel scaffolds and artificial bones and skin tissues. Our group successfully constructed one type of RHC, derived from the cell adhesion domain of type I collagen, and achieved large-scale production in an *E. coli* expression system. Preliminary tests verified its safety and reliability, showing good water solubility, which is beneficial for the post-processing of preparation techniques for multiform scaffolds.

Stem cell therapy not only requires the growth and proliferation of stem cells on scaffolds but also the promotion of stem cell differentiation in repairing bone defects. In fact, understanding how the directional differentiation of stem cells is achieved remains elusive to researchers (Xiao et al., 2019; Liu et al., 2020; Shang et al., 2020). During the course of hPDLSC differentiation into osteogenic cells and endochondral ossification, specific growth and differentiation factors are needed to induce bone formation. Bone morphogenetic proteins (BMPs) are widely used in bone tissue engineering, including BMP-2 and transforming growth factor-β3 (TGF-β3) (Park et al., 2015). TGF-β3 has been used for cartilage repair, tissue regeneration, and wound healing *in vivo* (Liu et al., 2015). UgoRipamonti et al. confirmed that TGF-β3 significantly induced bone formation in primate baboon skull defect models and patients with segmental mandibular defects (Ripamonti et al., 2016a; Ripamonti et al., 2016b; Ripamonti et al., 2017). Our laboratory previously found that TGF-β3 facilitates the osteogenic differentiation of hPDLSCs *in vitro*. Based on the concept of bionic repair, we designed a hybrid TGF-β3/RHC/CS freeze-dried sponge (TRFS) for the loading of hPDLSCs (TRFS-h) to repair skull defects.

Although stem cell therapy has made considerable advances in animal experiments, it has a long way to go before its widespread use in clinical applications. For this reason, stem cell therapy is likely to remain a topic of interest in future studies. In the present study, the effects of RHC/CS scaffolds with hPDLSCs on the repair of critical-size skull injury in rats were studied with the aim of gaining insights into stem cell therapy for the development of novel therapeutic strategies in future studies.

## Materials and Methods

### Materials

CS was supplied by Zhengzhou Corey Fine Chemical (MW 30–36 kDa) (with 55% deacetylation), TGF-β3 and RHC were provided by Jinan University Biopharmaceutical R and D Center (Guangzhou, China), and α-modified minimum essential medium (α-MEM), fetal bovine serum (FBS), and trypsin-EDTA were purchased from Gibco BRL. Penicillin and streptomycin (P/S) were purchased from MD Bio, China. MTT was purchased from MP Biomedicals (United States). Antibodies against collagen-1 (COL Ⅰ) were purchased from Affinity Biosciences (Cincinnati, OH, United States), BMP-2 and RUNX2 were purchased from Bioss (Boston, MA, United States), and GAPDH and an horseradish peroxidase-conjugated secondary antibody were purchased from Cell Signaling Technology (Boston, MA, United States).

### Preparation and Characterization of TRFS

CS freeze-dried sponges were prepared as previously reported (Zang et al., 2014). Briefly, CS was dissolved in 1% (v/v) glacial acetic acid and lyophilized. It was placed in a 95% ethanol solution for 2 h, after which the ethanol was discarded. Next, the CS was immersed in a 10% sodium hydroxide solution for 2 h and repeatedly cleaned with deionized water until the pH was approximately 7. CFS was sterilized and stored for later use.

To obtain a suitable carrier, 1, 2, and 3% CFS were selected for optimization. The characteristics involved the water absorption rate, expansion rate, water vapor transmission rate, moisture retention, and porosity structure, which were calculated as reported previously (He et al., 2020). The surfaces of the freeze-dried sponges were coated with a thin layer of gold and observed using scanning electron microscopy (SEM) (XL30; Philips, Amsterdam, Netherlands).

The TGF-β3 freeze-dried sponges (TFS), RHC freeze-dried sponges (RFS), TGFβ3/RHC/CS freeze-dried sponges (TRFS) were prepared as follows: sterile CFS was immersed in sterile TGF-β3 (20 nmol/L) and RHC (1,000 nmol/L) with shaking overnight at 4°C; the freeze-dried sponges were fabricated by vacuum lyophilization.

### Degradation of TRFS *in vitro* and Profile of TGF-β3 Release From TRFS

Next, the degradation rate of CFS was determined. Briefly, dried CFS of weight ^0^W was added to a 50 ml centrifuge tube. Then, 50 ml of phosphate-buffered saline containing 20 μg/ml lysozyme was added and incubated at 37°C. Samples of each group were collected at 1, 2, 3, 4, 5, 6, 7, and 8 weeks and freeze-dried for weighing (Wt). The degradation rate was determined using the following formula:

D%=(W0−Wt)/W0×100%.

The release of TGF-β3 from TFS was measured using an ELISA kit (Cusabio, Wuhan, China). Briefly, the scaffold (three replicates/group) was placed in a 1.5 ml Eppendorf tube, and then 1 ml of minimum essential medium (MEM) was added, followed by incubation at 37°C for 360 h. Then, 1 ml of MEM was collected and 1 ml of fresh MEM was added at 1, 12, 48, 72, 168, and 360 h. The samples were stored at −80°C until measurement. ELISA was performed according to the manufacturer’s instructions. Light absorbance was read using a microplate reader (Thermo Lab Systems, Waltham, MA, United States) at a wavelength of 450 nm.

### hPDLSC Growth and Osteogenic Differentiation in TRFS

#### hPDLSC Growth in TRFS

The cytotoxicity of TRFS was evaluated using an extraction test (Li et al., 2019). Briefly, hPDLSCs were cultured in a 96-well plate at a density of 1 × 10 ^4^ cells/well in MEM and 10% FBS for 24 h. The cells were treated with 25, 50, 75, or 100% TRFS extract. The positive and negative groups were treated with 5% dimethyl sulfoxide and normal medium, respectively. After 24 and 48 h of culture, the MTT assay was used to evaluate the results.

The growth of hPDLSCs in TRFS was observed using SEM. The prepared cell suspension was added to the front and back sides of the freeze-dried sponges. Before observation, the sample was subjected to gold sputtering using a gold spray carbonator and observed using SEM (XL30; Philips).

To further study cell growth in TRFS, hPDLSCs (2.0 × 10^5^ cells/mL) were cultured in TRFS for 3 days and then stained with calcein AM/PI (Calcein-AM/PI Double Stain Kit; Shanghai, China), followed by fluorescence microscopy (LSM700; Zeiss, Jena, Germany) to observe the staining by detecting red (535 nm, AM) and green (490 nm, PI) fluorescence.

#### hPDLSC Osteogenic Differentiation in TRFS

hPDLSCs were seeded in 24-well plates containing CFS, RFS, TFS, and TRFS at a density of 5 × 10^4^ cells/well. Then, hPDLSCs were cultured in osteogenic differentiation medium consisting of α-MEM containing 10^–8^ M dexamethasone (Sigma-Aldrich, St. Louis, MO, United States), 10 mM ß-glycerophosphate (Sigma-Aldrich), 50 ng/ml ascorbic acid (Sigma-Aldrich), 10% FBS, and 1% penicillin-streptomycin. The osteogenic medium was changed every 2 days. After cultivation for 3, 7, and 14 days, the freeze-dried sponge was stained with an alkaline phosphatase (ALP) staining kit (Beyotime Institute of Biotechnology, Shanghai, China) to evaluate their osteogenic differentiation capacity.

### *In vivo* Studies of Skull Defect SD Rat Model

All SD rats (male, 8 weeks old, 220 ± 20 g) used in this study were purchased from the animal center of Guangdong province (no. 44007200069979). Animals were caged under controlled room temperature, humidity, and light (12/12-h light-dark cycle) with access to water and food ad libitum. The experimental protocols used in this study were approved by the Institutional Animal Care and Use Committee of Jinan University (approval no. 2019228). Experiments were conducted according to the guidelines for animal care and use of China and were approved by the Animal Ethics Committee of the Chinese Academy of Medical Science.

An extreme skull defect model of SD rats was established to evaluate the repair properties of TRFS. SD rats (*n* = 6 per group) were randomly assigned to three groups (A) CFS group (B) TRFS group, and (C) TRFS loaded with hPDLSCs group (TRFS-h). TRFS-h was prepared as follows: hPDLSCs were seeded in TRFS at a density of 5 × 10^4^ cells/well and cultured in osteogenic differentiation medium for 14 days. Then, the rats were anesthetized with an intraperitoneal injection of 2% sodium thiopental (40 mg/kg), and the hair in the skull region was shaved. Skull defects were generated on both sides of the rat skull (5 mm in diameter) using a medical dental drill. Subsequently, the implants of freeze-dried sponges were placed onto the defect on the right side as the experimental group, whereas the defect on the left side was considered as a model group without any implants. The animal skin was sutured, and the edge of the wound was sterilized. Penicillin (40,000 units/day) was administered for 3 days after the operation to prevent infection. The feeding, activity, and wound infection of the rats were monitored daily. At 12 weeks post-surgery, six mice were randomly selected from each group for micro-computed tomography (CT) examination. After observation, the animals were anesthetized and sacrificed by cervical dislocation to obtain excisional biopsies of the implant area, including sufficient normal surrounding area for paraffin section treatment, hematoxylin-eosin (HE) and Masson staining, and immunohistochemical analysis.

### Micro-CT Examination

Animals selected at 12 weeks were evaluated with a micro-CT 80 scanner (Scanco Medical, Bassersdorf, Switzerland) for the analysis of new bone formation within defects. Three-dimensional (3D) reconstruction was then performed using micro-CT scanning to evaluate the reconstruction of skull defects. Bone volume (BV) and bone volume to total volume ratio (BV/TV) were analyzed using Mimics 17 software. BV/TV, indicating the proportion of mineralized tissue, was calculated for comparison.

### Histochemical Staining

Briefly, the calvaria tissue was fixed with 4% paraformaldehyde and embedded in paraffin for histological sectioning. After dewaxing and hydration, the sections were examined with HE staining to analyze the formation of new bone. Masson’s trichrome staining was performed to further analyze the formation of collagen. The sections were analyzed, and images were captured using a microscope (Olympus IX71; Tokyo, Japan).

In addition, the sections were used for immunohistochemical analysis to evaluate the expression of Runx2, BMP-2, and COL I. Lastly, the sections were dehydrated and sealed for microscopic observation (Olympus IX71).

### Subcutaneous Implant Surgical Procedure

Kunming (KM) mice (male, 5 weeks old, 22 ± 2 g) used in this study were purchased from the animal center of Guangdong province (no. 44007200070156). The feeding conditions were the same as described above. Animals were anesthetized by intraperitoneal injection of 2% sodium pentobarbital (40 mg/ml). The dorsal area of the KM mice was shaved, and the animals were placed in the prone position on an aseptic operating platform. Longitudinal skin incisions with a maximum length of 1 cm were created along the dorsum of the mice. Within each incision, the subcutaneous space was dissected and irrigated with 0.9% sterile saline to expose each pocket. The materials were prepared in advance and implanted into the skin pocket, and the incisions were sutured. Animals were housed with free access to food and water and were administered 40,000 units of penicillin per day for 3 days post-surgery. At 4, 8, and 12 weeks post-surgery, the animals were sacrificed by cervical dislocation. The implanted materials were removed and evaluated for material degradation. The heart, liver, spleen, lung, and kidney of the rats were dissected 4, 8, and 12 weeks after subcutaneous implantation and examined with HE staining.

### Statistical Analysis

All data are expressed as the mean ± standard deviation (SD) of at least three independent experiments. Statistical analyses were performed using GraphPad Prism 6 software (GraphPad Software Inc, La Jolla, CA, United States). Differences between more than two groups were analyzed using one-way ANOVA followed by Tukey’s HSD comparison test. Statistical significance was set at *p* < 0.05.

## Results

### Optimization of Chitosan Concentration in Freeze-Dried Sponges

**FIGURE 1:**
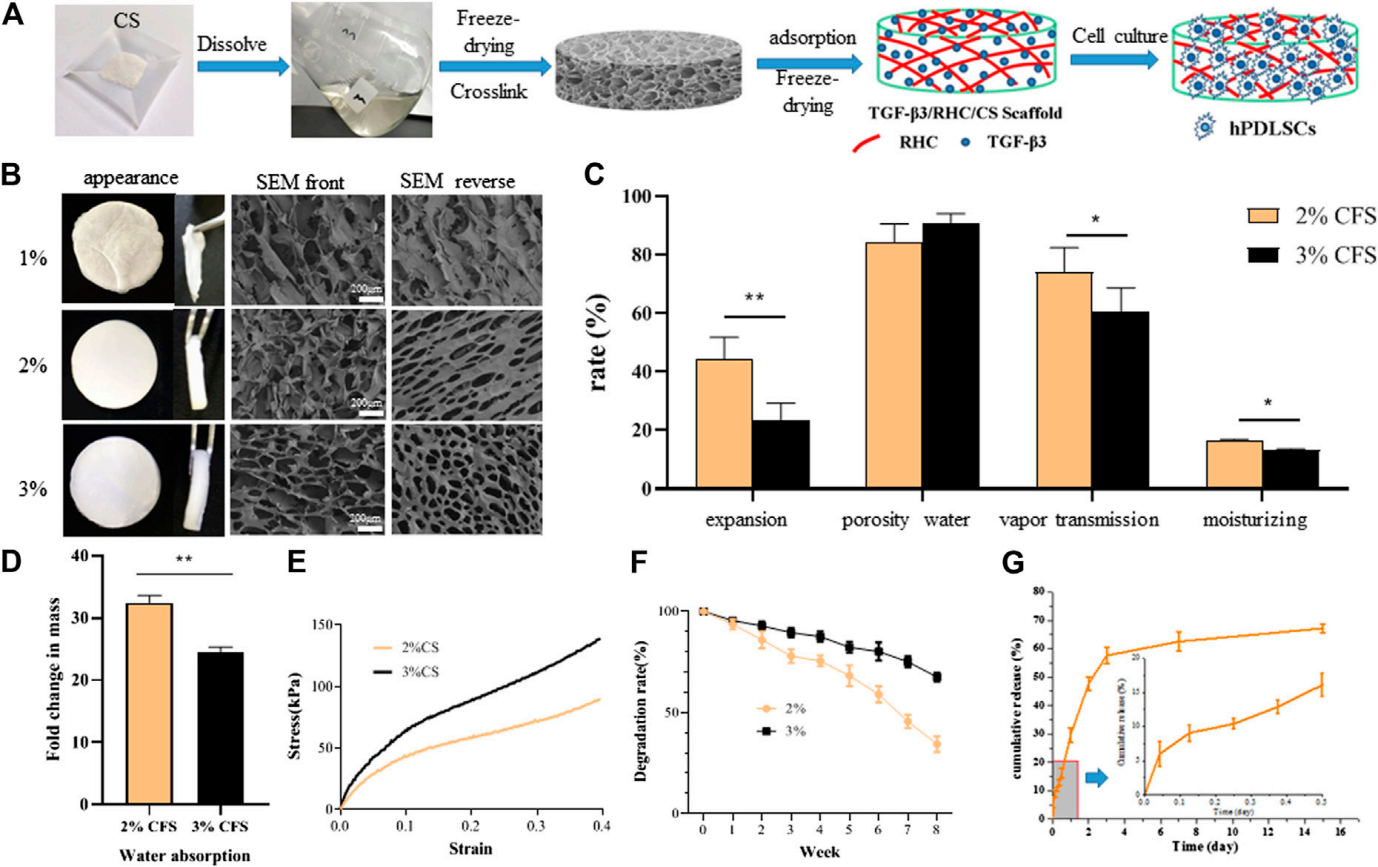
Preparation and physicochemical properties of CFS **(A)** CFS preparation process **(B)**. SEM observation of freeze-dried sponge morphology **(C)** Expansion rate test; Porosity of freeze-dried sponge, Water vapor transmittance test and Moisture retention test **(D)** Water absorption capacity test (*n* = 5, *p < 0.05, **p < 0.01 *vs* 2% CS) **(E)** Mechanical properties **(F)** Degradation experiment **(G)** release curve of TGF-β3 from 2% freeze-dried sponge.

The freeze-dried sponge preparation process is shown in Figure 1A. The CFS was characterized by the following indices: porosity, mechanical properties, water absorption, swelling degree, water vapor transmission, and moisture retention. Figure 1B shows that the 1% CFS structure collapsed without obvious pore formation. Therefore, subsequent studies on 1% CFS were not conducted. The 2 and 3% CFS had a porous three-dimensional structure and formed a staggered interconnected honeycomb structure on the front and a staggered network pore structure on the back. The surface of 2% CFS had ovoid pores with a pore size of 143 ± 33.1 μm, which is significantly higher than that of 3% CFS (87.6 ± 39.4 μm, *p* < 0.01). In Figure 1C, the expansion rate of 2% CFS was greater than 40% which was significantly different from that of 3% CFS (*p* < 0.01). Moreover, 2% CFS had better water vapor permeability (*p* < 0.05) than 3% CFS and better moisture retention rate (16.25 ± 1.37%) than 3% CFS (13.18 ± 0.97%) (*p* < 0.01). The porosity of 2% CFS was 84.9 ± 5.9% and that of 3% CFS was 90.2 ± 3.7%, which were not significantly different (*p* > 0.05). It can be seen from Figure 1D that the 2% CFS can absorb more than 30 times its own weight in aqueous solution, which is significantly different from that of the 3% CFS (*p* < 0.01). Figure 1E shows that 2% CFS is approximately 90 kPa when the strain is equal to 40%, while the 3% CFS hydrogel is approximately 138 kPa, indicating that 2% sponge has lower mechanical properties than 3% sponge (*p* < 0.01).

In summary, while 3% CFS had slightly higher mechanical properties, 2% CFS had better water absorption, water vapor permeability, and moisture retention. As such, 2% CFS was selected for the preparation of TRFS and subsequent studies. TRFS was prepared from 2% CS and had the characteristics of 2% CS, including a porous three-dimensional structure with high water vapor permeability and moisture retention.

### Degradation of TRFS *in vitro* and Profile of TGF-β3 Release From TRFS

Figure 1F shows the *in vitro* degradation of TRFS. TRFS gradually degraded by approximately 65% at week 8. As shown in Figure 1G, TGF-β3 was stably released from TRFS. After a short initial burst (16.08%), TGF-β3 from the TRFS scaffold was released in a sustained manner, and the release rate decreased with time. By day 15, approximately 71.28% of the total TGF-β3 was released. This result suggested that the TRFS scaffold was a promising delivery carrier for TGF-β3 and allowed for a controlled release.

### TRFS Promotes the Proliferation and Osteogenic Differentiation of hPDLSCs

#### TRFS Promotes the Proliferation of hPDLSCs *in vitro*

**FIGURE 2:**
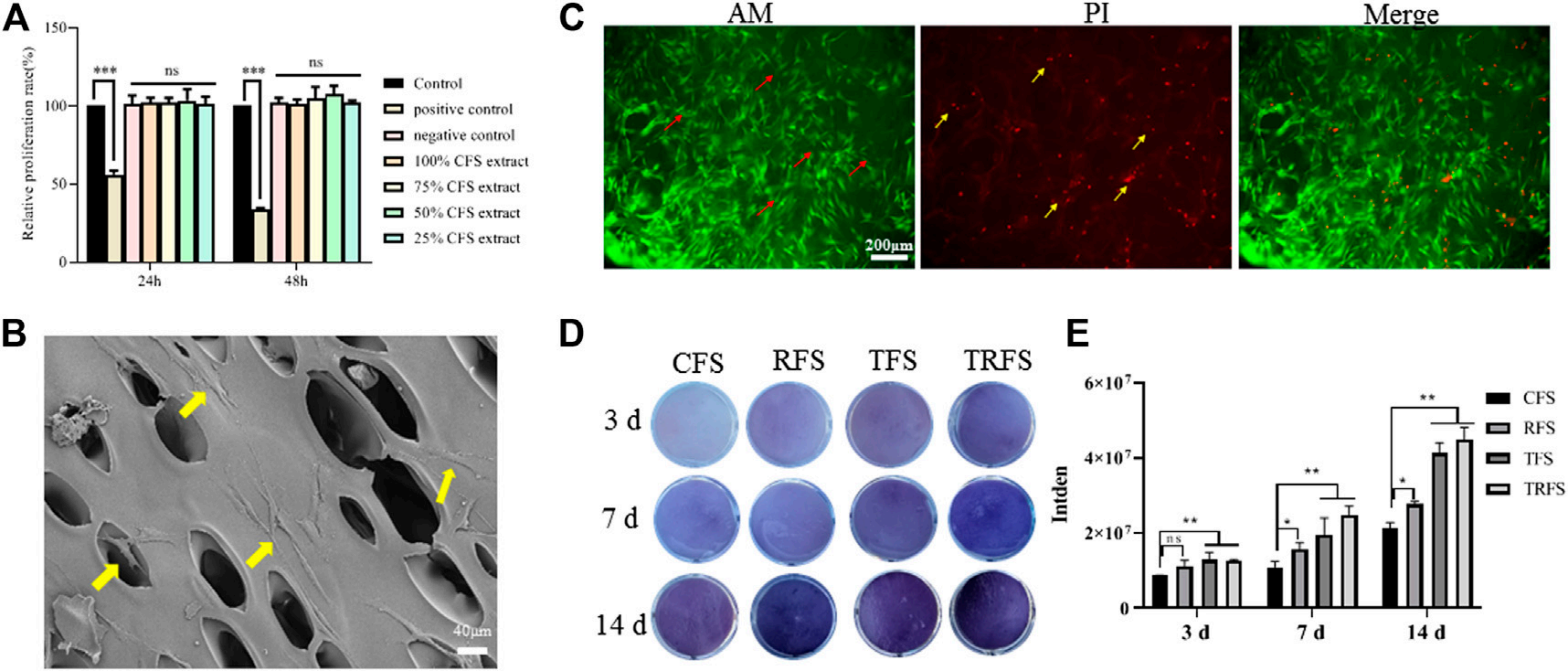
Cell growth and osteogenic differentiation on TRFS **(A)** Detection of TRFS extract effect on the growth of hPDLSCs using MTT assay (*n* = 6, *p < 0.05, **p < 0.01, ***p < 0.001, ns means no significant difference *vs* control) **(B)** The growth of hPDLSCs on TRFS was observed using SEM **(C)** Calcein-AM/PI double staining was used to observe the survival of hPDLSCs cultured on TRFS for 7 days. Red arrows indicate living cells, and yellow arrows indicate dead cells **(D)** ALP staining (purple) was used to detect the ALP activity of hPDLSCs in CFS, RFS, TFS, and TRFS after 3, 7, and 14 days of osteogenic induction **(E)** Quantitative analysis of color depth (*n* = 3, *p < 0.05, **p < 0.01, ns means no significant difference *vs* CFS).

According to the results of the MTT *in vitro* cytotoxicity test (Figure 2A), there was no significant difference between the TRFS extract group and the control group (*p* > 0.05). Figure 2B shows the SEM image of hPDLSCs cultured in TRFS for 3 days hPDLSCs showed good adhesion and extension states on the surface of the material. Figure 2C shows the results of calcein-AM/PI double staining after hPDLSCs were cultured on TRFS for 7 days. The results showed that hPDLSCs grew well on TRFS with complete cell structure, and the number of living cells was far more than that of dead cells, indicating that TRFS is suitable for proliferation of hPDLSCs. These results indicated that TRFS was beneficial for cell growth.

#### TRFS Facilitates the Osteogenic Differentiation of hPDLSCs

hPDLSCs were cultivated in CFS, RFS, TFS, and TRFS, respectively, and the ALP values were determined at 3, 7, and 14 days after osteoblastic induction (Figure 2D). We observed significant differences between the TRFS and CFS groups at 3, 7, and 14 days (*p* < 0.05, *vs.* control; Figure 2D). The TFS and TRFS groups showed noticeably higher ALP levels than the CFS group. The results indicated that TRFS provided the most beneficial environment for hPDLSC differentiation.

### TRFS Promotes Skull Defect Repair

**FIGURE 3:**
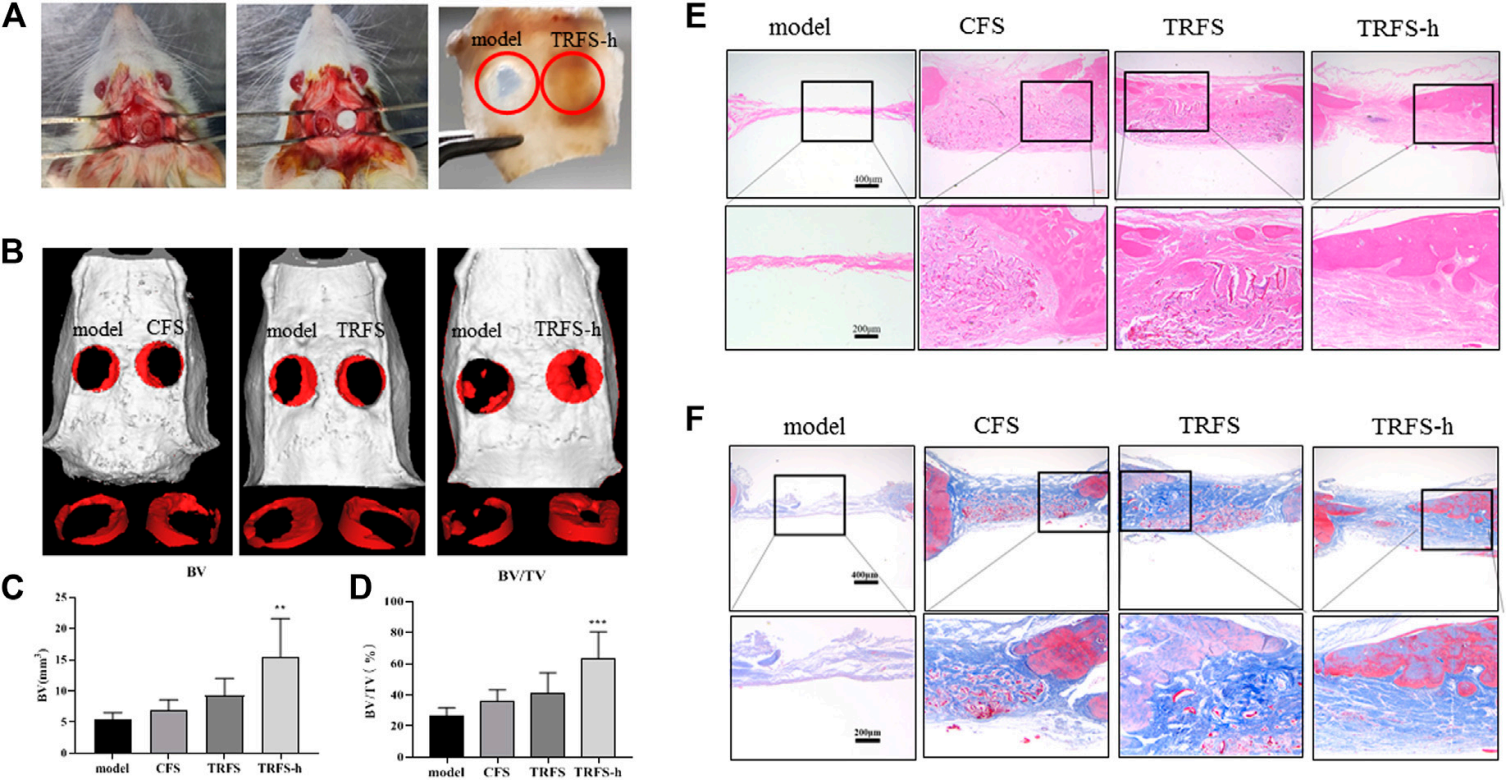
TRFS containing hPDLSCs was used to repair skull injury in SD rats **(A)** Establishment and material implantation of ultimate skull defect (5 mm) model in rats **(B)** Micro-CT scanning and three-dimensional reconstruction of skull defects in SD rats **(C)** Analysis of new bone mass **(D)** Analysis of bone volume at the site of skull defect **(E)** HE staining of skull tissue after 12 weeks **(F)** Masson staining of skull tissue after 12 weeks (*n* = 6, **p < 0.01, ***p < 0.001 *vs* model).

Figure 3A shows the material implantation of the SD rat skull defect and the skull at 12 weeks post-surgery. The material implanted on the right side of the defect was noticeably filled with tissue, while the defect area on the left of the model was only a transparent film. As shown in Figure 3B, at 12 weeks after modeling, the effect of repairing bone defects in the TRFS-h group was significantly better than that in the other groups. In the TRFS-h group, a large amount of new bone formed, and the defect area was significantly reduced. By contrast, in the model, CFS and TRFS groups, only a few new bone tissues formed at the edge of the bone defect after 12 weeks Figure 3C shows the statistical results for the new BV at the defect site. The BV in the TRFS-h group was significantly higher than that in the model group at 12 weeks (*p* < 0.01). Figure 3D shows the new BV fractions. The BV fraction of the TRFS-h group was higher than that of the other groups at 12 weeks and significantly higher than that of the model group (*p* < 0.01).

HE staining of the skull specimen at 12 weeks (Figure 3E) showed that, in the model group, no new bone formation was found at the bone defect site, and only a small amount of tissue fiber filling was observed. In the TRFS-h group, there was significantly more new bone formed at the bone defect site than that in the other groups. The material in the bone defect was completely degraded, and a large amount of fibrous tissue was observed at the bone defect site with no inflammatory cell infiltration. The Masson staining results (Figure 3F) showed that the bone defect in the model group only had a small amount of blue fibrous tissue filling, and no noticeable new bone formation in the bone defect site was observed. In the CFS, TRFS, and TRFS-h groups, compared with the model group, a large amount of blue fibrous tissue was found to fill the bone defect, and undegraded material was surrounded by blue fibrous tissue. The new bone tissue grew from the edge to the inside of the bone defect. The material was completely degraded, and many new blood vessels grew in the fibrous tissue of the bone defect, with no inflammatory cell infiltration.

### TRFS Promotes the Expression of Osteogenesis-Associated Proteins

**FIGURE 4:**
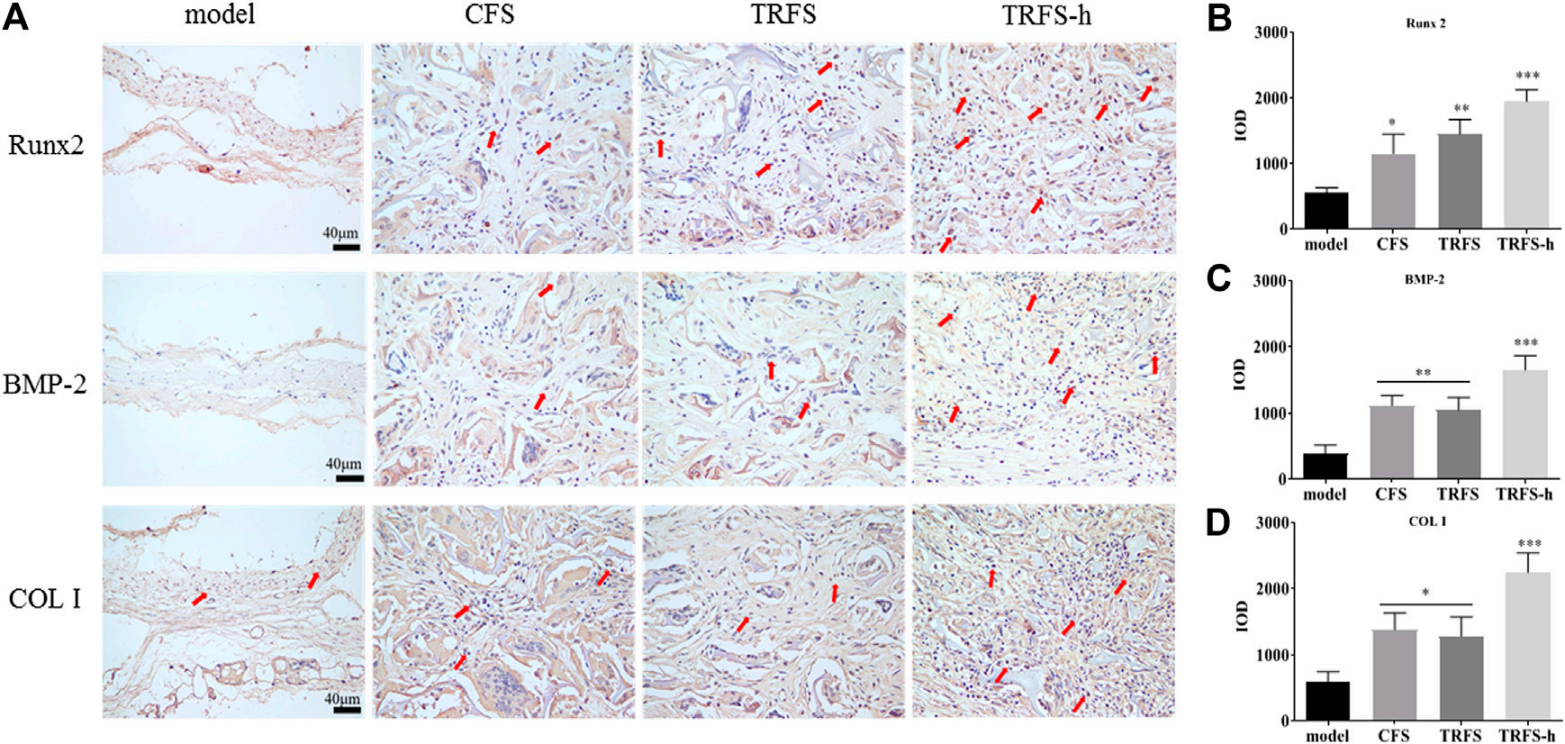
Osteogenesis-associated protein immunohistochemical staining was performed in skull tissue sections at 12 weeks post-surgery **(A)** Immunohistochemical staining of osteogenesis-associated proteins Runx2, BMP-2, and COL I. Red arrows indicate positive cells **(B)** Runx2 positive statistics **(C)** BMP-2 positive statistics **(D)** COL I positive statistics (*n* = 3, *p < 0.05, **p < 0.01, ***p < 0.001 *vs* model).

Figure 4A shows the Runx2, BMP-2, and COL I immunohistochemical staining of skull tissue sections at 12 weeks. In the model group, only a few cells showed positive expression reactions in bone defects. In the experimental groups, positive cell reactions were observed around the material, indicating that the material promoted the expression of osteogenesis-associated proteins in cells at the bone defect site. Among them, the number of positive reaction cells around the TRFS-h group was significantly higher than that in the other groups. The statistical results indicated that there was a significant difference between the experimental groups and the model group (Figures 4B–D; *p* < 0.05). In the TRFS-h group, the expression of Runx2, BMP-2, and COL I was significantly higher than that in the model group (*p* < 0.001), indicating that TRFS-h promoted osteoblast activity.

### Degradation of TRFS *in vivo*

**FIGURE 5:**
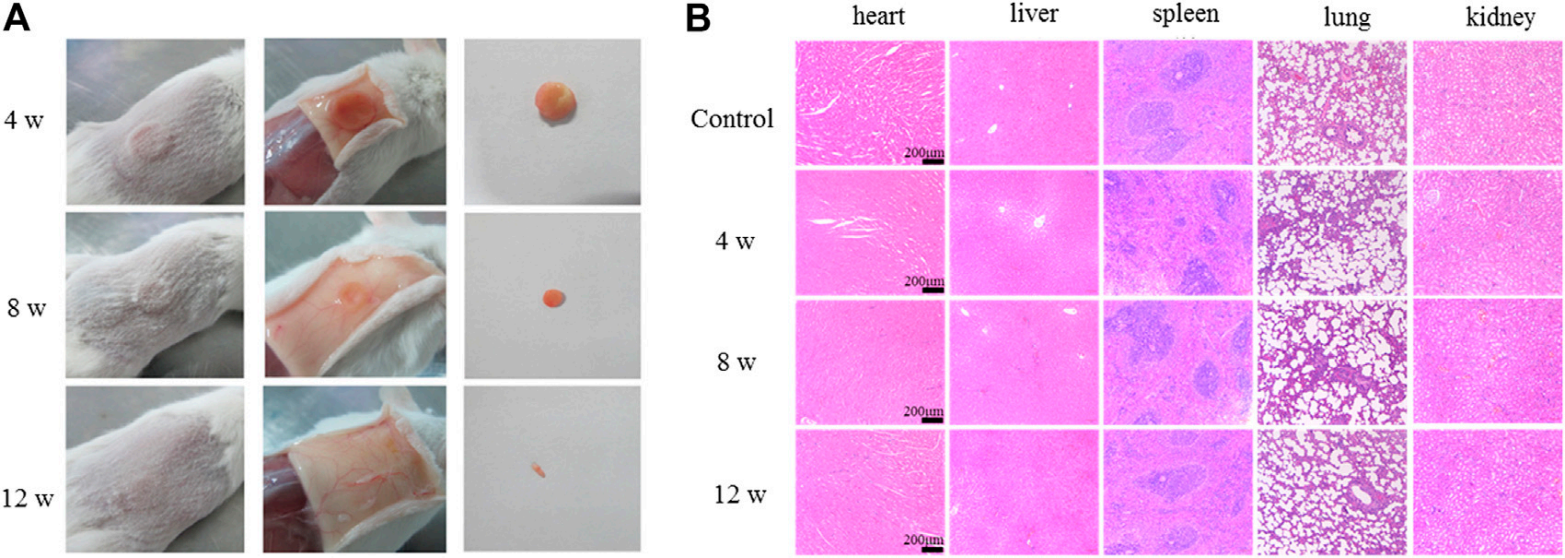
Degradation of TRFS *in vivo* **(A)** Study on degradation of TRFS *in vivo* at 4, 8, and 12 weeks post-implantation into the dorsal subcutaneous tissue of male KM mice **(B)** HE staining of the heart, liver, spleen, lung, and kidney (*n* = 10).

The degradation of the materials was observed at 4, 8, and 12 weeks (Figure 5A). The results showed that TRFS could be degraded gradually, and approximately, one-fourth was degraded at 8 weeks, and the degradation was basically complete at 12 weeks. The heart, liver, spleen, lung, and kidney of the rats were dissected 4, 8, and 12 weeks after subcutaneous implantation. The tissues were converted to paraffin sections and observed after HE staining. Compared with the tissue sections of control rats, there were no significant differences in those of experimental rats (Figure 5B).

## Discussion

Scaffold materials play a vital role in regenerative medicine and are bridges connecting seed stem cells and regenerative tissues. They provide structural and spatial support for the adhesion, proliferation, and differentiation of stem cells and serve as a template to guide tissue regeneration (Pérez-García et al., 2019; Zhai et al., 2020). In bone tissue engineering, scaffolds facilitate the delivery of biofactors and sustain mechanical forces until the regenerated tissue can bear them. Therefore, many factors need to be considered when designing scaffolds for the craniofacial region, including porosity, biocompatibility, degradability, surface morphology, and mechanical strength (Shakya and Kandalam, 2017). In this context, we used the vacuum freeze-drying method to design a hybrid TRFS to load hPDLSCs for the repair of calvarial defects in rats. We evaluated the main characteristics of CFS prepared with different CS concentrations, including apparent morphology, pore size, porosity, water absorption, expansion rate, moisture retention, water vapor transmission rate, mechanical properties, and *in vitro* degradation. Various reports have previously highlighted that pores smaller than 300 µm encourage endochondral ossification (Karageorgiou and Kaplan, 2005; Murphy et al., 2010). In our study, 2% CFS formed a porous three-dimensional sponge structure with higher porosity and more regular pores, interpenetrating the entire scaffold. They were found to be conducive to cell migration, nutrient transmission, and metabolite discharge. Biodegradation is an important criterion for the design of bone tissue engineering (BTE) scaffolds. The implanted BTE scaffolds should undergo degradation over time and should match the formation of new bone (Saravanan et al., 2016). The 2% CFS was gradually degraded by approximately 65% at week 8 *in vitro* and degraded almost completely after 12 weeks *in vivo*, which is consistent with the bone repair cycle. Overall, 2% CFS was more suitable for the physical and chemical properties required for calvarial defect repair and thus was selected for further study.

Numerous reports have shown that CS-based materials not only exhibit the excellent activities of CS but also show other appealing performance of combined materials, giving rise to good synergistic properties of CS and its composite materials, such as RHC (Andonegi et al., 2020). RHC is a biological protein formed by high-density fermentation and separation processes through genetic engineering technology. RHC not only retains the excellent properties of collagen, but also has a high water solubility and processability and low immunogenicity compared to native collagen, without the risk of virus infection. It has been applied to various groups of biological scaffold materials. Cell adhesion is the primary condition for cell proliferation, migration, and differentiation. Crystalline violet staining and cytoskeleton staining showed that RHC effectively promoted the adhesion and spread of hPDLSCs (Supplementary Figure S1). Deng et al. reported that electrospun RHC/CS nanofibers crosslinked *in situ* are ideal candidates for use in wound healing applications (Deng et al., 2018). Yang et al. prepared recombinant collagen sponges loaded with BMP-2 as bone graft substitutes for the treatment of spinal instability caused by degenerative spondylosis (Yang et al., 2004). Zhou et al. successfully prepared a three-dimensional porous scaffold based on nanohydroxyapatite/RHC/poly (lactic acid) (nHA/RHC/PLA), which had the main component and hierarchical microstructure similar to that of natural bone. The nHA/RHC/PLA scaffold facilitated the healing of segmental bone defects and exhibited good osteoconductivity (Zhou et al., 2017). The hybrid RHC/CS scaffold also increased the adhesion of cells and promoted hPDLSC osteogenic differentiation, bone regeneration, and skull defect repair.

With advances in research, this ubiquitous polypeptide has been found to play an important role in bone remodeling and the regulation of bone differentiation. TGF-β3, an important regulatory molecule of MSCs, affects the proliferation, renewal, and differentiation of stem cells (Valdez et al., 2012). (Klar et al., 2013) found that TGF-β3 induced endochondral ossification by regulating BMP activity, thereby inducing bone formation (Klar et al., 2014). Deng et al. (2017) found that TGF-β3 may initiate the process of bone regeneration by recruiting endogenous stem cells. In a previous study, our research group found that TGF-β3 did not affect the proliferation of hPDLSCs but promoted their migration and differentiation into bone *in vitro*. To further demonstrate the effects of the above *in vivo*, a critical size skull defect SD rat model was used. Zhang et al. (2020) created two round defects on both sides of the rat skulls and implanted a gelatin-hydroxyapatite composite cryogel to investigate the regeneration efficiency of rat calvarial defects. Lam et al. (2019) transplanted MSCs loaded with polycaprolactone microvectors into rat skull defects to enhance bone healing in rat calvarial defects. After the operation, the rats showed normal physiological activities, such as eating and excretion, and no fatalities were recorded among the animals for the duration of the experiment. In the present study, the imaging and histological results at 12 weeks after surgery showed that few new bone tissues formed in the model group, and large defects remained. In the TRFS-h group, a large amount of new bone formation was observed, and the defect area was significantly reduced. Micro-CT was used to continuously observe skull defects in the experimental rats. Micro-CT is a non-traumatic detection tool with high resolution and good reconstruction accuracy and can continuously detect live animals. Three-dimensional reconstruction and analysis of the internal bone tissue structure can be performed to analyze the dynamic changes in sample bone density and mass. The results showed that the TRFS-h group significantly promoted the repair of rat skull defects. The volume and mass of new bone in the TRFS-h group were significantly higher than those in the other groups (*p* < 0.05). The results of the TRFS subcutaneous implantation experiment showed that TRFS did not cause noticeable inflammation in the body and degraded almost completely after 12 weeks, which is consistent with the bone repair cycle.

Based on the above experimental results, we further explored the mechanism by which TRFS promoted stem cell osteogenesis *in vivo*. The results of HE staining showed that after 12 weeks, the new bone morphology parameters at the bone defect site in the TRFS-h group were significantly higher than that in the other groups. A large number of thick new bones grew from the edge of the bone defect to the inside of the bone defect, and the materials in the bone defect were completely degraded. The Masson staining results showed that the bone defect in the TRFS-h group was filled with a large amount of blue fibrous tissue, and the undegraded sponge was surrounded by blue fibrous tissue. Further, the expression of Runx2, COL I, and BMP-2 was observed. Runx2 is the most specific and earliest expressed marker of osteoblasts. It is expressed by mesenchymal cells at the initial stage of bone development and exists throughout the differentiation process of osteoblasts (Zhang et al., 2003; Chen et al., 2014). Runx2 can control the transcription of many genes expressed on osteoblasts and is a necessary factor for osteoblast differentiation *in vivo* and *in vitro*. COL I is the main ECM protein secreted by the osteoblasts. It is the earliest product of osteoblasts in the formation of the bone matrix and is a characteristic marker of the osteoblast phenotype. BMP-2 is one of the most important cell signaling molecules that promotes bone formation, induces osteoblast differentiation, and plays an important role in bone formation. The results of immunohistochemical staining showed that the positive reactions of Runx2, COL I, and BMP-2 in the TRFS-h group were significantly higher than those in the other groups, indicating that the osteoblasts of the TRFS-h group were differentiated actively. Therefore, the above results indicate that TRFS loaded with hPDLSCs can effectively repair rat skull injuries.

PDLSCs have been proposed as the most promising cells for the regeneration of severely damaged PDL tissue, among other stem cells such as dental pulp stem cells, stem cells from human exfoliated deciduous teeth, and dental follicle stem cells (Tsumanuma et al., 2011). hPDLSCs have the ability to differentiate into osteoblasts, chondrocytes, cementoblasts, and adipocytes, as well as myocytes and neural cells (Tomokiyo et al., 2008; Song et al., 2012). Professor Qingsong Ye, the director of Wuhan University Regenerative Medicine Center, is the head of a research group that focuses on the clinical application and transformation of odontogenic stem cells. Ye et al. prepared bioactive hydrogels combined with odontogenic stem cells to repair large space peripheral nerve injury and found that odontogenic stem cells could directly differentiate into new nerve tissue to repair the defect site (Luo et al., 2021). Many researchers are currently trying to develop innovative and critical methods for bone defect therapy from various perspectives to improve health and quality of life (Maeda, 2020). Both human dental pulp mesenchymal stem cells (hDPMSCs) and hPDLSCs originate from odontogenic tissue. An hDPMSC injection was recently approved for use in humans after a clinical trial (CXSL1700137), encouraging us to continue our research.

## Data Availability

The raw data supporting the conclusion of this article will be made available by the authors, without undue reservation.

## Abbreviations

CS, chitosan; CT, computed tomography; hPDLSCs, human periodontal ligament stem cells; MSC, mesenchymal stem cells; RHC, recombinant human-like collagen; SEM, scanning electron microscopy; TGF, transforming growth factor; TRFS, TGF-β3/RHC/CS freeze-dried sponge; TRFS-h, TRFS loading hPDLSCs.
