# Supplementary figures and images for: Transforming Growth Factor-β3/Recombinant Human-like Collagen/Chitosan Freeze-Dried Sponge Primed With Human Periodontal Ligament Stem Cells Promotes Bone Regeneration in Calvarial Defect Rats
Source: Front Pharmacol. 2021 Apr 23;12:678322. doi: 10.3389/fphar.2021.678322 (PMC8103166; doi:10.3389/fphar.2021.678322)

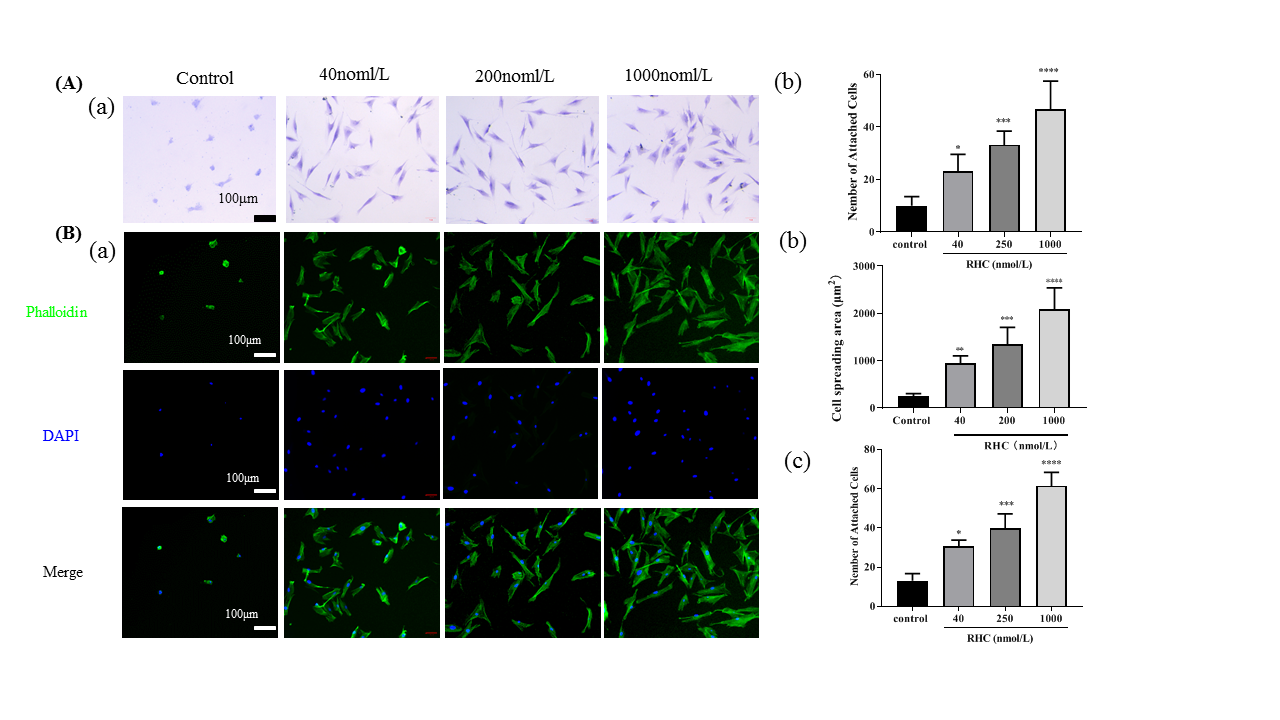

Supplement: Supplementary file 1 [file Image1.TIF]
